# Fast and efficient synthesis of microporous polymer nanomembranes via light-induced click reaction

**DOI:** 10.3762/bjoc.13.54

**Published:** 2017-03-17

**Authors:** Qi An, Youssef Hassan, Xiaotong Yan, Peter Krolla-Sidenstein, Tawheed Mohammed, Mathias Lang, Stefan Bräse, Manuel Tsotsalas

**Affiliations:** 1 Institute of Functional Interfaces (IFG), Karlsruhe Institute of Technology (KIT), Hermann-von-Helmholtz-Platz 1, D-76344 Eggenstein-Leopoldshafen, Germany; 2 Zewail City of Science and Technology, Center for Materials Science, Sheikh Zayed District, 6th of October City, 12588, Giza, Egypt; 3 Institute of Physics and Technology, International X-ray Optics Lab, National Research Tomsk Polytechnic University (TPU), 30 Lenin ave., Tomsk 634050, Russia; 4 Institute for Organic Chemistry (IOC), Karlsruhe Institute of Technology (KIT), Fritz-Haber-Weg 6, D-76131 Karlsruhe, Germany,; 5 Institute of Toxicology and Genetics (ITG), Karlsruhe Institute of Technology (KIT), Hermann-von-Helmholtz-Platz 1, D-76344 Eggenstein-Leopoldshafen, Germany

**Keywords:** click chemistry, conjugated microporous polymers (CMPs), microporous materials, nanomembranes, thin films, thiol–yne coupling reaction (TYC)

## Abstract

Conjugated microporous polymers (CMPs) are materials of low density and high intrinsic porosity. This is due to the use of rigid building blocks consisting only of lightweight elements. These materials are usually stable up to temperatures of 400 °C and are chemically inert, since the networks are highly crosslinked via strong covalent bonds, making them ideal candidates for demanding applications in hostile environments. However, the high stability and chemical inertness pose problems in the processing of the CMP materials and their integration in functional devices. Especially the application of these materials for membrane separation has been limited due to their insoluble nature when synthesized as bulk material. To make full use of the beneficial properties of CMPs for membrane applications, their synthesis and functionalization on surfaces become increasingly important. In this respect, we recently introduced the solid liquid interfacial layer-by-layer (LbL) synthesis of CMP-nanomembranes via Cu catalyzed azide–alkyne cycloaddition (CuAAC). However, this process featured very long reaction times and limited scalability. Herein we present the synthesis of surface grown CMP thin films and nanomembranes via light induced thiol–yne click reaction. Using this reaction, we could greatly enhance the CMP nanomembrane synthesis and further broaden the variability of the LbL approach.

## Introduction

The synthesis of microporous organic and inorganic materials such as zeolites [1], mesoporous silica [2] as well as metal-organic frameworks (MOF) [3–4] and covalent organic frameworks (COF) [5–7] attracted large attention because of their high potential in catalysis, gas storage and separation as well as in organic electronics [8]. Among the microporous materials, conjugated microporous polymers (CMPs) [9–10] or porous aromatic frameworks (PAF) [11] have favorable properties for many applications, since they combine a high chemical and thermal stability, which is comparable to inorganic materials, with the variability of organic compounds.

Nevertheless, their insoluble nature has so far greatly limited their processing and integration into functional devices, since CMPs and PAF are usually synthesized as highly crosslinked interconnected and insoluble powders [12]. Only few examples of soluble and therefore processable CMP materials are known, all limited to linear CMPs [13–14].

To overcome the issue of low processability, recently the group of Jiang and our group introduced the interfacial synthesis using an electro-activated approach [15] and a copper catalyzed azide–alkyne cycloaddition (CuAAC) approach, respectively [16]. These procedures are still limited to conductive substrates or associated with long reaction times.

In this work, we present a novel strategy for the LbL synthesis of CMP thin films and nanomembranes, using the light-induced and catalyst-free thiol–yne coupling (TYC) reaction.

TYC has gained large attention as a representative of the click chemistry concept [17]. In the TYC reaction, usually a photoinitiator creates thiyl radicals [18–20], which react with nearby alkyne moieties to form covalent sulfur–carbon bonds and vinyl radicals. Additional thiol moieties can undergo hydrogen transfer to the vinyl radical leading to thiyl radicals and vinyl sulfides. The vinyl sulfides can then undergo a thiol–ene coupling (TEC) reaction, leading to bis-sulfide species. TYC has been used for surface modification [21–22], biofunctionalization [23–24] and fabrication of 3D structures via direct laser writing (DLW) [25].

## Results and Discussion

### Synthesis of CMP thin films

We prepared the CMP nanomembranes in a LbL approach using the thiol–yne coupling (TYC) reaction. In order to perform the reaction on surfaces, we first functionalized the substrates with an alkyne terminated self-assembled monolayer, which presents initial groups for the stepwise growing of the CMPs using the TYC reaction.

In the first step, we immersed the functionalized surface in a solution of the tetra-topic thiol building block (tetrakis(4-sulfanylphenyl)methane, TPM-SH) and a small amount of photoinitiator (2-hydroxy-1-[4-(2-hydroxyethoxy)phenyl]-2-methylpropan-1-one) [26]. Afterwards we irradiated the substrate using a standard UV lamp at a wavelength of 365 nm for 3 minutes. We then rinsed the substrate thoroughly with absolute THF and immersed the substrate in a solution of the tetra-topic alkyne building block (tetrakis(4-ethynylphenyl)methane, TPM-alkyne), again with a small amount of photoinitiator. Then we irradiated the substrate for 3 minutes and rinsed the substrate thoroughly with absolute THF.

**Figure 1:**
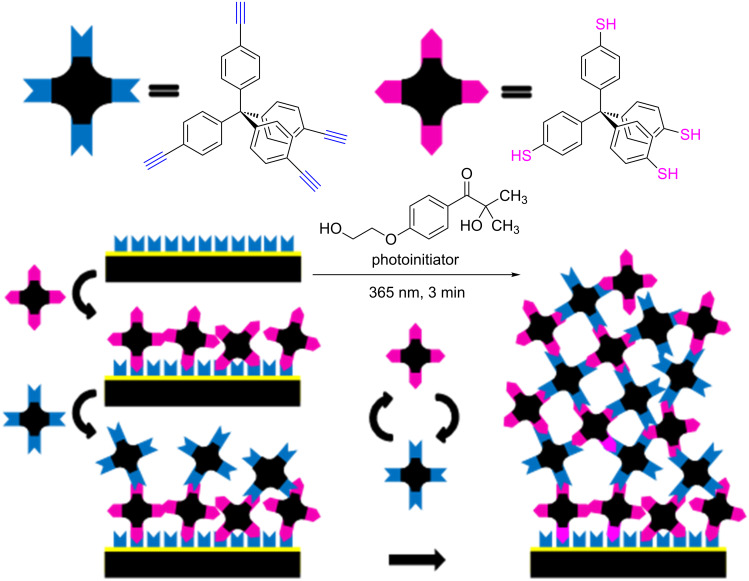
LbL synthesis with TPM-SH and TPM-alkyne using light-induced TYC reaction in the presence of the photoinitiator 2-hydroxy-1-[4-(2-hydroxyethoxy)phenyl]-2-methylpropan-1-one.

Figure 1 shows the LbL synthesis procedure as well as the molecular structures of the reactants used in the described reaction.

We repeated the described reaction cycle 20 times to obtain CMPs thin films on functionalized gold wafers.

### Characterization of CMP thin films

**Figure 2:**
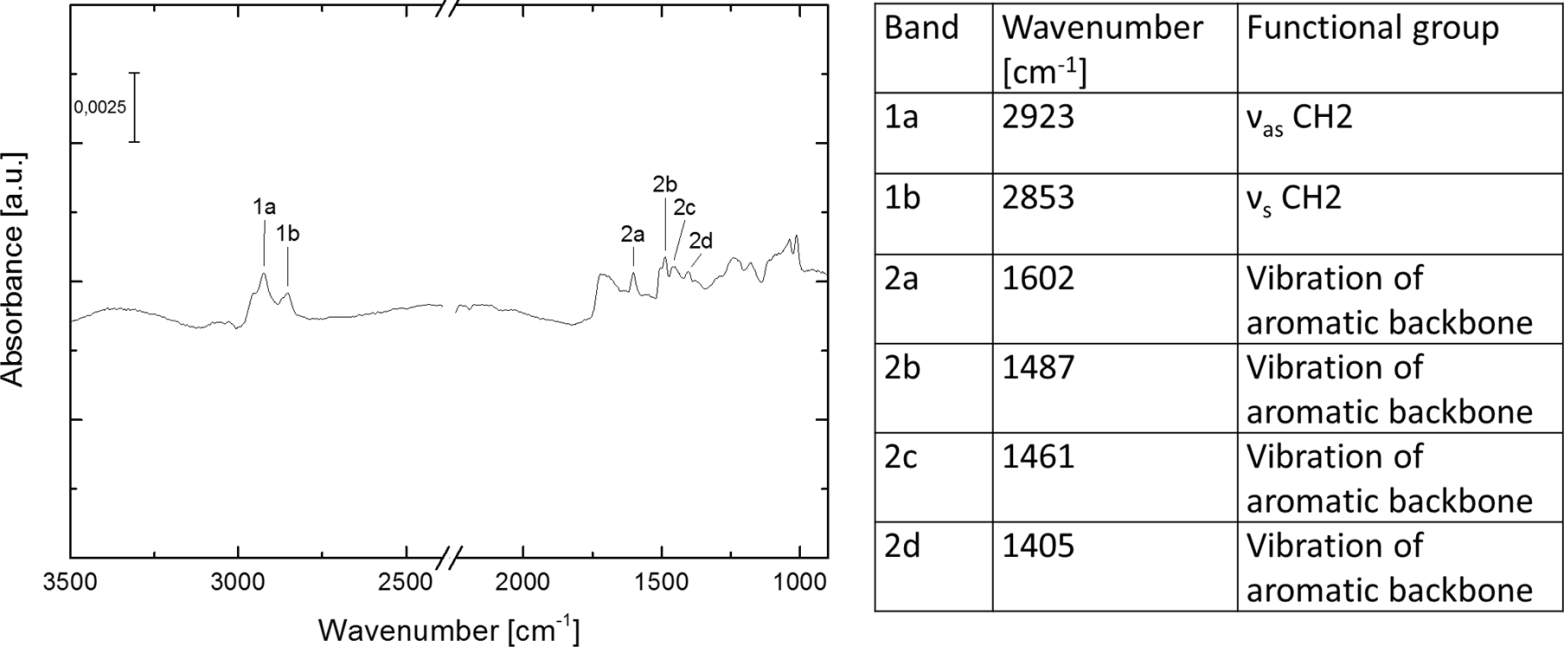
IRRA-Spectrum of the CMP thin film on a gold-coated silicon wafer and the corresponding band assignments.

We characterized the reaction using infrared reflection absorption spectroscopy (IRRAS). Figure 2 shows the IRRA spectrum of the CMP thin film after 20 reaction cycles and the corresponding band assignments.

The absence of bands associated to alkyne and thiol functional groups in the IRRA-spectra suggest an almost quantitative reaction. (For IRRA-spectra of the starting materials, see Supporting Information File 1, Figures S1–S3.)

**Figure 3:**
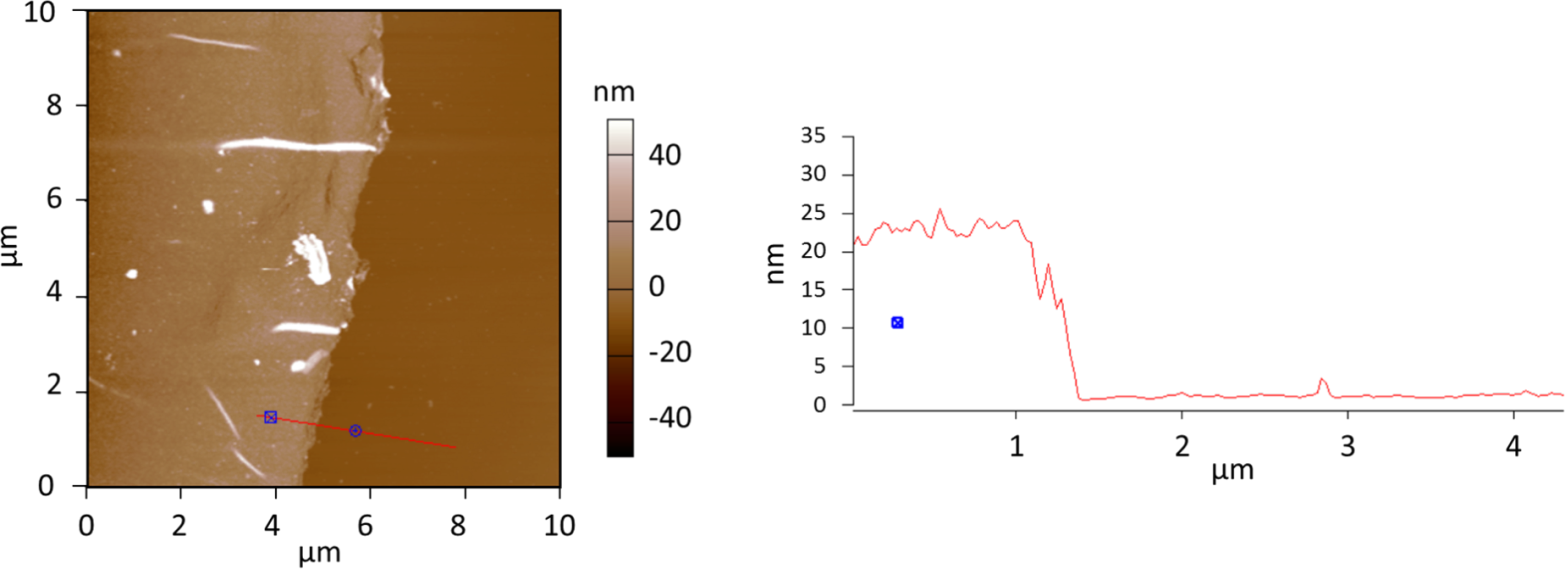
AFM image and line-scan across the edge of the CMP thin film.

We evaluated the thickness of the CMP thin film using ellipsometry. The measurements show an average thickness of about 25.1 ± 0.1 nm with a mean squared error (MSE) value of 5.69 after fitting with Cauchy mode with the parameters A_n_ = 1.399, B_n_ = 0.051, C_n_ = −0.0026, k-amplitude = 0 and exponent = 1.5 suggesting a very low surface roughness. To further confirm the thickness we performed the LbL synthesis on a sacrificial substrate [27–28]. Prior to the dissolution of the substrate, we coated the CMP thin film with a stabilizing layer of poly(methyl methacrylate) (PMMA). Upon substrate dissolution, we transferred the PMMA stabilized CMP thin film to a fresh gold substrate. After drying, we dissolved the PMMA layer in acetone, leaving only the CMP thin film. We then investigated the thickness of the film by an atomic force microscope (AFM) line scan along the edge of the film. Figure 3 shows the AFM image and the line-scan across the edge of the CMP thin film.

The AFM investigation also suggests a homogeneous thickness of the CMP thin film and the line-scan across the edge confirms a thickness of roughly 20 nm after 20 reaction cycles. The growth rate of roughly 1 nm per reaction cycle is in the same order as the previously described LbL synthesis of CMP nanomembranes using CuAAC click chemistry [16].

### Synthesis of freestanding CMP nanomembranes

**Figure 4:**
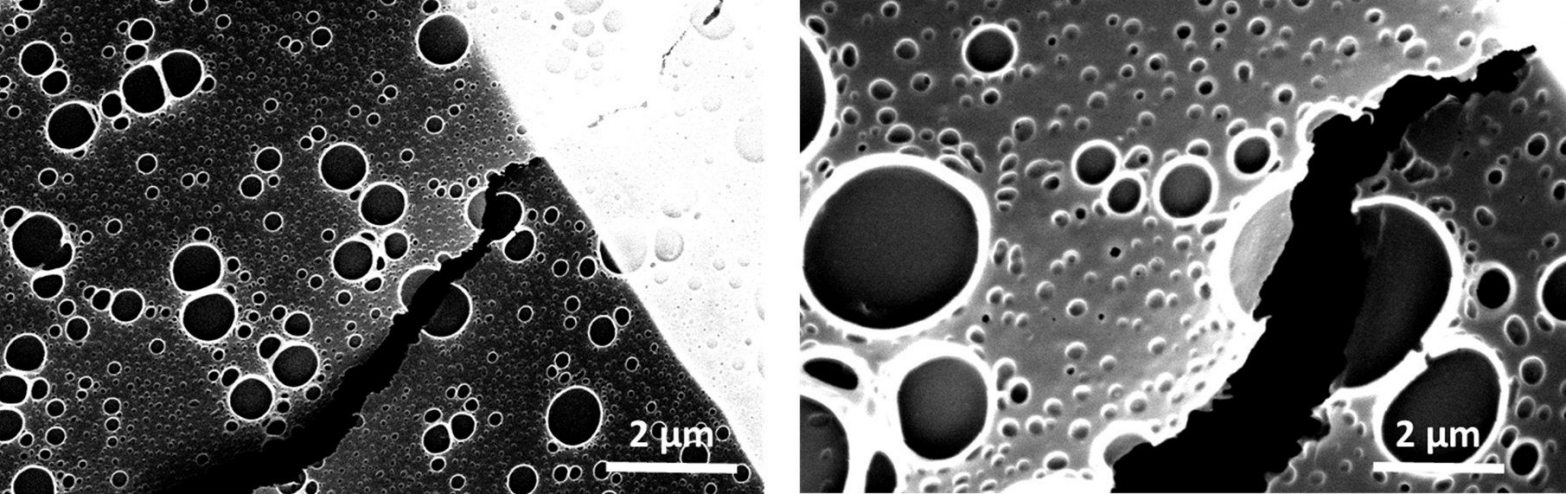
SEM images of freestanding CMP nanomembranes coated with a stabilizing PMMA layer containing large holes. The SEM images confirm the homogeneous thickness and freestanding nature of the CMP nanomembranes synthesized via TYC reaction.

In order to produce freestanding CMP nanomembranes, we coated the CMP thin films on sacrificial substrates with a stabilizing layer of PMMA containing large holes [29]. This stabilizing layer was spin-coated from a dichloromethane (DCM) solution containing PMMA and polystyrene (PS) in a weight ratio of PMMA/PS = 4:1. During spin-coating, the PS phase separates into islands, which then were selectively dissolved using cyclohexane. Afterwards we dissolved the sacrificial substrate to obtain the freestanding CMP membranes. To investigate the freestanding CMP nanomembrane we transferred it to a copper grid and recorded scanning electron microscopy (SEM) images. Figure 4 shows the SEM images of the CMP nanomembrane.

## Conclusion

We described a new synthesis of CMP thin films and nanomembranes using a thiol–yne coupling (TYC) reaction. The TYC reaction allows a rapid synthesis of homogeneous thin films with a thickness of about 1 nm per reaction cycle as confirmed by ellipsometry and AFM investigations. The thin films show high mechanical stability as evidenced by the possibility to create feestanding membranes across holes of about 3–5 µm diameter. The rapid and scalable synthetic method for CMP nanomembranes described in this article, along with the possibility to transfer the nanomembranes to virtually any support, allows the integration of TYC based CMP materials in functional devices for applications in organic electronics or gas and liquid phase separation.

## Experimental

**Chemicals**: All chemicals were purchased from commercial sources and used without further purification if not stated otherwise. Cyclohexane, dichloromethane and dry tetrahydrofuran (THF) were purchased from Merck Millipore; acetone was purchased from VWR Chemicals. Dry THF was degassed three times via freeze-pump-thaw prior to use. PMMA average *M*_w_ ≈120.000, PS average *M*_w_ ≈170.000, 2-hydroxy-1-[4-(2-hydroxyethoxy)phenyl]-2-methylpropan-1-one), iodine and potassium iodide were purchased from Sigma-Aldrich. TPM-SH [30] and TPM-alkyne [31] were synthesized as described in the literature.

**Substrates**: The sacrificial substrate consists of a 150 nm gold film on mica. For analytical measurements, we transferred the membrane to a Si(100) wafer, coated with 5 nm titanium and 100 nm gold (Au/Ti/Si). The substrates were obtained from Georg-Albert-PVD, Germany and stored under an argon atmosphere prior to use.

**Infrared reflection absorption spectroscopy (IRRAS)**: The IRRA-spectra were recorded on a Bruker Vertex 80 purged with dried air. The IRRAS accessory (A518) has a fixed angle of incidence of 80°. The data were collected on a middle band liquid nitrogen cooled MCT detector. Perdeuterated hexadecanethiol-SAMs on Au/Ti/Si were used for reference measurements [32]. The absorption band positions are given in wavenumbers 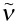 (cm^−1^).

**Scanning electron microscopy (SEM):** We recorded SEM images using a FEI Philips XL30 (FEI Co., Eindhoven, NL), a field emission gun environmental scanning electron microscope (FEG-ESEM). Samples have been coated with a thin layer (about 5 nm) of a gold/palladium film in order to avoid charging and improve samples conductivity. All specimen were imaged under high-vacuum conditions (1.0 Torr), using an acceleration voltage of 20 keV.

**Atomic force microscopy (AFM):** AFM-imaging was performed using an Asylum Research Atomic Force Microscope, MFP-3D BIO. The AFM was operated at 25 °C in an isolated chamber in alternating current mode (AC mode). AFM cantilevers were purchased from Ultrasharptm MikroMasch. Three types of AFM-cantilevers were used, an NSC-35 (resonance frequency 315 kHz; spring constant 14 N/m), an NSC-36 (resonance frequency: 105 KHz; spring constant: 0.95 N/m) and an NSC-18 (resonance frequency: 75 kHz; spring constant: 3.5 N/m).

**Self-assembled monolayer (SAM) preparation:** For SAM formation, a clean gold substrate (2.2 cm × 2.2 cm) was rinsed with absolute ethanol and then immersed in a solution of *S*-[11-oxo-11-(propargylamino)undecyl] thioacetate (AcSC_10_H_20_C(O)NHCH_2_C≡CH) (with a concentration of 1 mmol/L) in ethanol for 18 h. Afterwards the substrate was taken out, rinsed thoroughly with ethanol and dried in a nitrogen stream [33].

**Preparation of conjugated microporous polymer (CMP) films:** 6.7 mg of TPM-SH (15.0 µmol, 1.00 equiv), 6.3 mg of TPM-alkyne (15.0 µmol, 1.00 equiv) and 1.1 mg of 2-hydroxy-1-[4-(2-hydroxyethoxy)phenyl]-2-methylpropan-1-one) (5 µmol, 0.333 equiv) as photoinitiator were separately dissolved in 20 mL abs. THF. The synthesis was carried out under inert conditions using an argon atmosphere. At first, 1 mL of TPM-SH solution and 0.5 mL of the photoinitiator solution were added to the SAM coated substrate and stirred gently to ensure proper mixing. Then the mixture was exposed to 365 nm UV-light for 3 minutes. Afterwards, the substrate was rinsed with dry THF. Subsequently, 1 mL of TPM-alkyne solution and 0.5 mL of the photoinitiator solution were added to the substrate and stirred gently to ensure proper mixing. Then the mixture was exposed to 365 nm UV-light for 3 minutes. Then, the substrate was again rinsed with dry THF. The procedure was then repeated for the next reactant 20 times each. After the cycles were completed, the wafer was taken out of the inert environment, washed thoroughly with dry THF and ethanol and dried using a nitrogen stream.

**Transfer of CMP nanomembranes:** To obtain freestanding nanomembranes, the CMP-films were grown on sacrificial substrates using the above-described procedure. The membrane was then obtained by following a procedure described in literature [28]. First, PMMA was spin coated as a supporting layer. Then, the mica was removed by floating in solutions of I_2_/KI/H_2_O; KI/H_2_O and in the last step by immersing the substrate in H_2_O. The retaining gold film was etched in a solution of I_2_/KI/H_2_O. The membrane was washed 3 times with water [28]. Then the membrane was transferred to Cu-TEM grids. The obtained membrane size was 0.3 cm × 0.3 cm.

**Preparation of freestanding nanomembranes:** To obtain freestanding nanomembranes, the CMP-films were grown on sacrificial substrates using the above-described procedure. The membrane was then obtained by following a procedure described in literature [29]: First PMMA/PS was spin coated as a supporting layer and afterwards rinsed overnight in cyclohexane to remove the PS. Then, the mica was removed by floating in solutions of I_2_/KI/H_2_O; KI/H_2_O and in the last step by immersing the substrate in H_2_O. The retaining gold film was etched in a solution of I_2_/KI/H_2_O. The membrane was washed 3 times with water [28]. Afterwards the membrane was transferred to either a glass slide or a gold coated Si-wafer. The obtained membrane size was 2 cm × 2 cm.

## Supporting Information

File 1 Additional IRRA spectra.
