# Supplementary material for: Fast and efficient synthesis of microporous polymer nanomembranes via light-induced click reaction
Source: Beilstein J Org Chem. 2017 Mar 17;13:558–63. doi: 10.3762/bjoc.13.54 (PMC5372710; doi:10.3762/bjoc.13.54)
Supplement: File 1 — Additional IRRA spectra. [file Beilstein_J_Org_Chem-13-558-s001.pdf]

Supporting Information

for

Fast and efficient synthesis of microporous polymer nanomembranes via light-induced click reaction

Qi An[1], Youssef Hassan[1,2], Xiaotong Yan[1], Peter Krolla-Sidenstein[1], Tawheed Mohammed[1,3], Mathias Lang[4], Stefan Bräse[4,5] and Manuel Tsotsalas*[1,4]

Address: [1]Institute of Functional Interfaces (IFG), Karlsruhe Institute of Technology (KIT), Hermann-von-Helmholtz-Platz 1, D-76344 Eggenstein-Leopoldshafen, Germany, [2]Zewail City of Science and Technology, Center for Materials Science, Sheikh Zayed District, 6th of October City, 12588, Giza, Egypt, [3]Institute of Physics and Technology, International X-ray Optics Lab, National Research Tomsk Polytechnic University (TPU), 30 Lenin ave., Tomsk 634050, Russia, [4]Institute for Organic Chemistry (IOC), Karlsruhe Institute of Technology (KIT), Fritz-Haber-Weg 6, D-76131 Karlsruhe, Germany, and [5]Institute of Toxicology and Genetics (ITG), Karlsruhe Institute of Technology (KIT), Hermann-von-Helmholtz-Platz 1, D-76344 Eggenstein-Leopoldshafen, Germany

Email: Manuel Tsotsalas - manuel.tsotsalas@kit.edu

* Corresponding author

Additional IRRA spectra

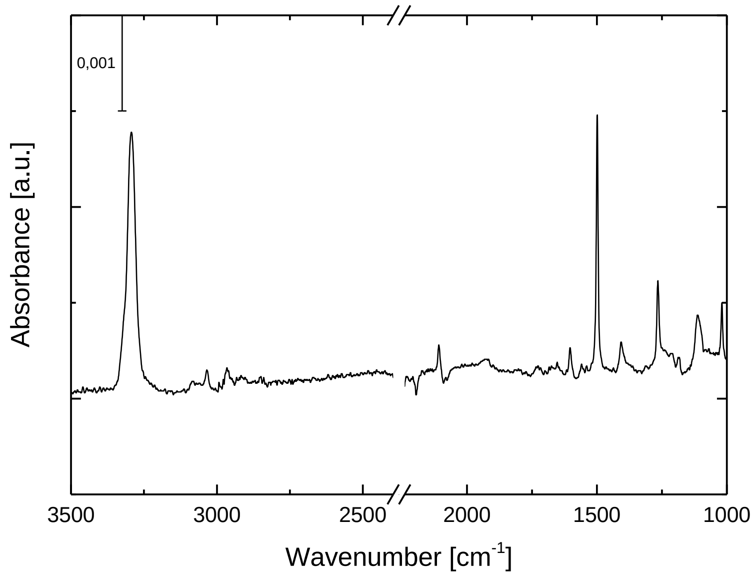

Figure S1: IRRA spectrum of Tetrakis(4-ethynyl-phenyl)methane (TPM-alkyne).

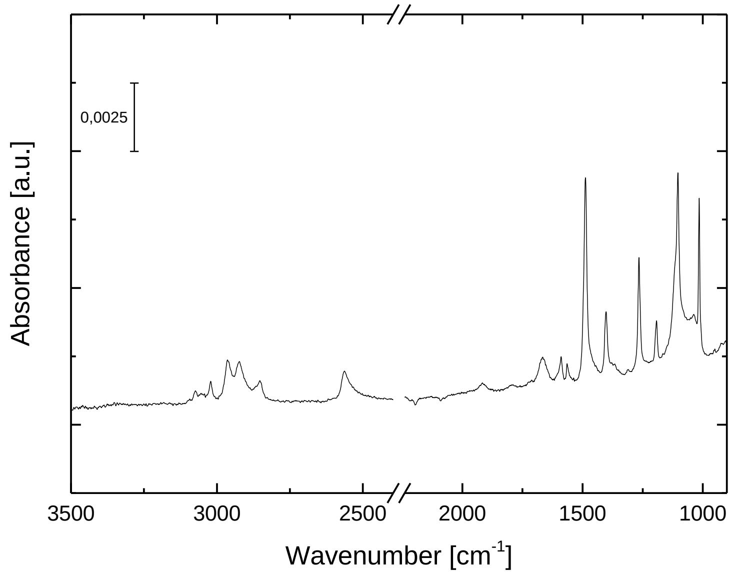

Figure S2: IRRA spectrum of Tetrakis(4-thiolphenyl)-methane (TPM-SH).

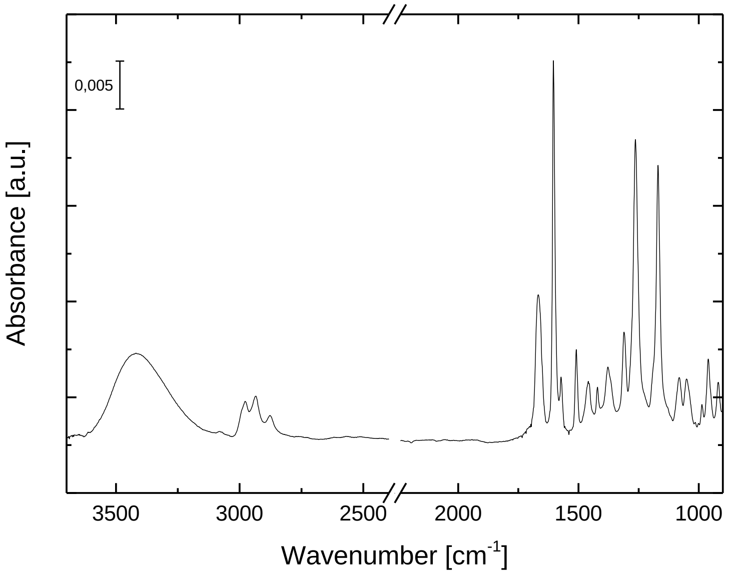

Figure S3: IRRA spectrum of 2-hydroxy-4'-(2-hydroxyethoxy)-2-methylpropiophenone.
